# MRI detection of brain abnormality in sickle cell disease

**DOI:** 10.1080/17474086.2021.1893687

**Published:** 2021-06-07

**Authors:** Hanne Stotesbury, Jamie Michelle Kawadler, Dawn Elizabeth Saunders, Fenella Jane Kirkham

**Affiliations:** Developmental Neurosciences Section, UCL Great Ormond Street Institute of Child Health, London, UK

**Keywords:** Sickle cell, mri, neuroimaging, neuroradiology, biomarkers

## Abstract

**Introduction**: Over the past decades, neuroimaging studies have clarified that a significant proportion of patients with sickle cell disease (SCD) have functionally significant brain abnormalities. Clinically, structural magnetic resonance imaging (MRI) sequences (T2, FLAIR, diffusion-weighted imaging) have been used by radiologists to diagnose chronic and acute cerebral infarction (both overt and clinically silent), while magnetic resonance angiography and venography have been used to diagnose arteriopathy and venous thrombosis. In research settings, imaging scientists are increasingly applying quantitative techniques to shine further light on underlying mechanisms.

**Areas covered**: From a June 2020 PubMed search of ‘magnetic’ or ‘MRI’ and ‘sickle’ over the previous 5 years, we selected manuscripts on T1-based morphometric analysis, diffusion tensor imaging, arterial spin labeling, T2-oximetry, quantitative susceptibility, and connectivity.

**Expert Opinion**: Quantitative MRI techniques are identifying structural and hemodynamic biomarkers associated with risk of neurological and neurocognitive complications. A growing body of evidence suggests that these biomarkers are sensitive to change with treatments, such as blood transfusion and hydroxyurea, indicating that they may hold promise as endpoints in future randomized clinical trials of novel approaches including hemoglobin F upregulation, reduction of polymerization, and gene therapy. With further validation, such techniques may eventually also improve neurological and neurocognitive risk stratification in this vulnerable population.

## 1. Introduction

**Figure 1.**
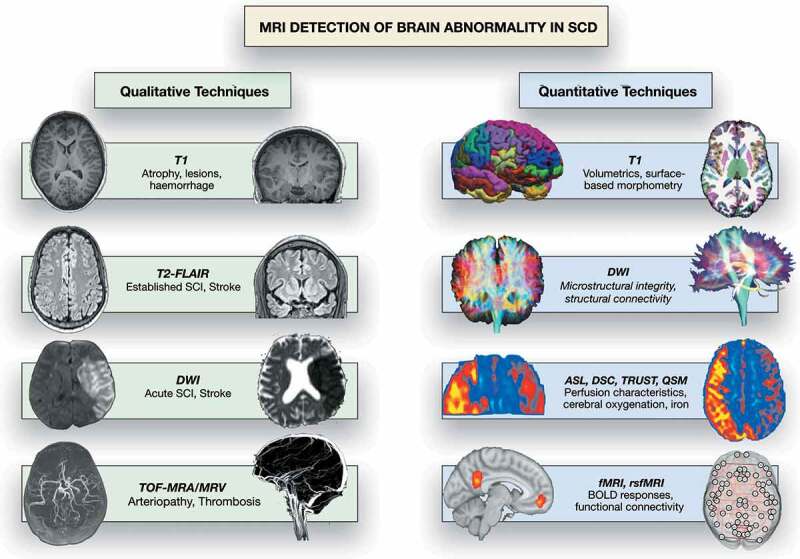
MRI detection of abnormality in SCD, showing common qualitative and quantitative MRI techniques that have yielded insight into neurological complications in SCD patients. DWI: diffusion-weighted imaging; TOF-MRA/V: Time of flight magnetic resonance angiography/venography; ASL: arterial spin-labeling; DSC: dynamic susceptibility contrast; TRUST: T2-relaxation under spin tagging; fMRI: functional MRI; rsfMRI: resting-state functional MRI; BOLD: blood oxygenation level dependent. DWI qualitative images from Hussain Z, Hilal K, Ahmad M, et al. (2 March 2018) Clinicoradiological Correlation of Infarct Patterns on Diffusion-weighted Magnetic Resonance Imaging in Stroke. Cureus 10(3): e2260. doi:10.7759/cureus.2260. MRV qualitative image from https://medpix.nlm.nih.gov/case?id=4510eec0-5199-4e4b-b803-15723ae51c31. rsfMRI connectivity image from Dr Jon Clayden

There is a broad spectrum of presentation with cerebrovascular accident (CVA) and other neurological complications in patients with sickle cell disease (SCD) [1–4]. Since the mid-1980s, magnetic resonance imaging (MRI) has been used to investigate brain pathology in SCD patients presenting with acute neurological symptoms and signs, in emergency and rehabilitation settings, and in observational cohort studies [1]. Techniques used clinically include T1-, T2-fluid attenuated inversion recovery (T2-FLAIR), T2*, susceptibility and diffusion weighted MRI, and time-of-flight angiography (MRA) and venography (MRV) (Table 1, Figure 1) The imaging has typically been interpreted qualitatively by neuroradiologists with variable degrees of specific training, using a number of different grading schemes and rating scales for MRI and MRA [5–13]. More recently, structural and hemodynamic quantitative approaches, including T1, diffusion, perfusion, quantitative susceptibility mapping (QSM), and functional MRI (Figure 1), in humans and in animal models of SCD interpreted by neuroscientists have allowed insight into potential mechanisms of neurological and neurocognitive compromise (Figure 1) [4,14–16]. The starting point for this review was a PubMed literature search of ‘magnetic’ or ‘MRI’ and ‘sickle’ over the past 5 years with screening for manuscripts related to the brain, finalized in June 2020 and supplemented with literature known to the coauthors.

**Table 1.** Overview of MRI techniques used in the detection of brain abnormalities in SCD, with a focus on current clinical and research methods. FLAIR: fluid-attenuated inversion recovery; DWI: diffusion-weighted imaging; DTI: diffusion tensor imaging; TOF-MRA/V: Time of flight magnetic resonance angiography/venography; ASL: arterial spin-labeling; DSC: dynamic susceptibility contrast. TRUST: T2-relaxation under spin tagging; fMRI: functional MRI; rsfMRI: resting-state functional MRI; BOLD: blood oxygenation level dependent

**MRI Technique**	**Current Clinical Uses in SCD** **(Qualitative)**	**Current Research Uses in SCD** **(Quantitative)**
**T1**	Detection of established atrophy, infarction, hemorrhage	Quantification of brain volumes and surface-based morphometry
**T2-FLAIR**	Detection of established overt infarction, silent cerebral infarction, posterior reversible encephalopathy syndrome	Quantification of infarct volumes
**DWI/DTI**	DWI: Detection of acute overt infarction, acute silent cerebral infarction	DWI: Quantification of acute infarct volumes, DTI: structural connectivity, and microstructural integrity
**TOF-MRA/MRV**	Detection of arteriopathy, Venous sinus thrombosis	-
**ASL/DSC**	-	ASL/DSC: Quantification of cerebral blood flow, transit and arrival times, and cerebrovascular reactivity
**TRUST**	-	Quantification of venous oxygen saturation and cerebral oxygen extraction fraction
**SWI/QSM**	SWI: Detection of hemorrhages and micro-hemorrhages	SWI: Quanitifcation of hemorrhages and micro- hemorrhages, QSM: Quantification of brain iron deposition, venous oxygen saturation and cerebral oxygen extraction fraction
**fMRI/rsfMRI**	-	fMRI: Quantification of blood-oxygenation-level dependent responses, cerebrovascular reactivity, rsfMRI: functional connectivity

## 2. Qualitative imaging in acute neurological settings

### 2.1. Clinical stroke

Without preventative strategies, clinical stroke, with focal signs lasting >24 hours, e.g. hemiparesis, abnormal gait including ataxia, dysphagia and visual field defect [1–4], is 250 times more common in children with SCD than in the general pediatric population [17], and commonly presents ‘out-of-the-blue’ in an apparently well child. Infarction is common in mid-childhood, between 2 and 10 years of age [18]. Stroke incidence decreases to a minimum between the ages of 20 and 29 years, but there is a further peak after the age of 35 [18]. Hemorrhage has the highest incidence in young adults (20–30 years) [18] but occurs in children [1–4,19]. The lifetime risk of stroke is at least 25%-30% [18].

### 2.2. Ischemic stroke

**Figure 2.**
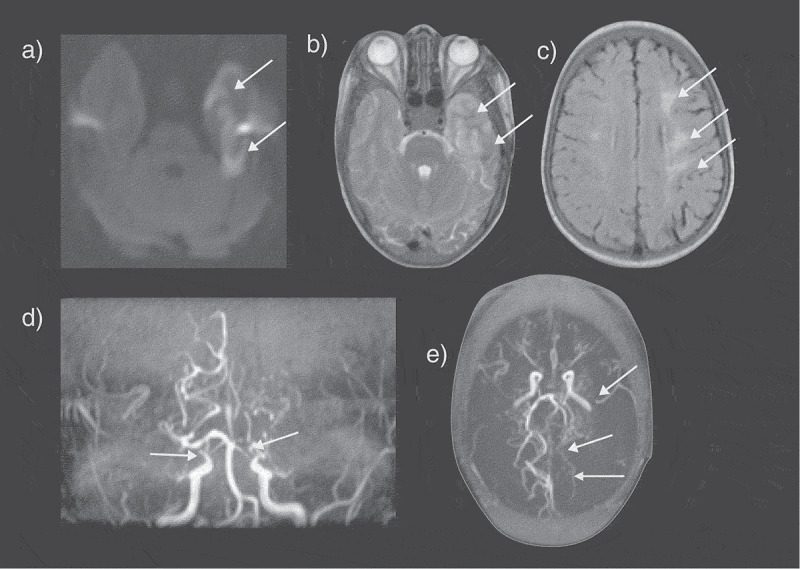
An SCD patient presented with seizures and a right sided neurological deficit (B) Axial T2-weighted images shows an acute left temporal lobe infarct in the left middle cerebral artery (MCA) territory with (A) restricted diffusion on the apparent diffusion map (ADC) map (arrows). (C) The FLAIR sequence revealed extensive mature ischemic changes within the subcortical, deep and periventricular white matter of the centrum semiovale, more marked on the left (arrows). (D) MRA revealed bilateral occluded terminal internal carotid arteries (ICA) and multiple moyamoya and pial collaterals (arrows). (E) The left posterior cerebral artery (PCA) is narrowed with distal pial vessels visible (arrows). FLAIR = fluid attenuated inversion recovery

**Figure 3.**
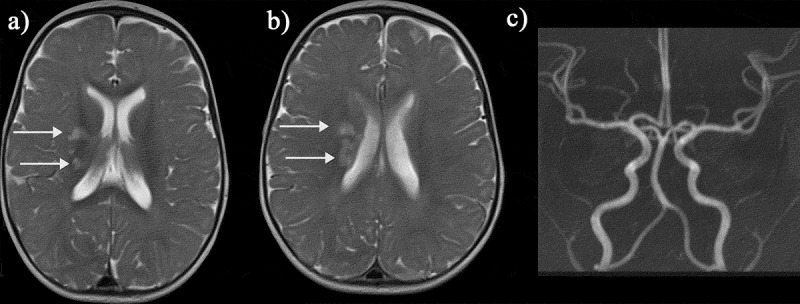
Basal ganglia infarct: This young child who is a compound heterozygote had a left hemiparesis at the age of six months, before transcranial Doppler screening was mandated. Prior to that, the child had had a cold on and off for the last few months and tended to breathe rather heavily and to snore on and off. (a,b) The T2-weighted magnetic resonance imaging showed patchy hyperintensity within the right superomedial striato-capsular region, with associated diffusion restriction (not shown), consistent with acute evolving infarction. (c) Magnetic resonance angiography was normal, as was imaging transcranial Doppler but patent foramen ovale was demonstrated on bubble contrast echocardiography although venography of the legs and pelvis did not demonstrate clot. Treatment was with aspirin as well as regular blood transfusion

**Figure 4.**
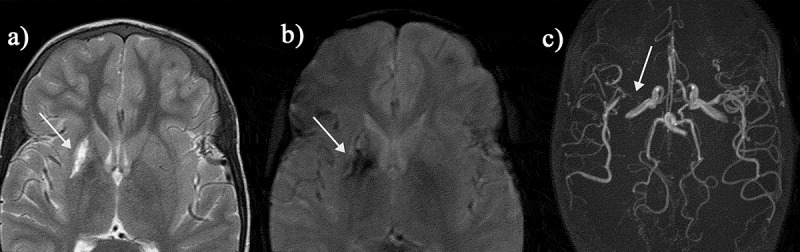
(a) Hemorrhagic basal ganglia infarct in a 16-yr-old girl not reporting symptoms but with asymmetric transcranial Doppler velocities (right time averaged mean of the maximum 39, left 164 cm/sec). The subtle hemorrhagic change is seen as a dark blush on the T2-weighted sequence but is well seen on the (B) T2* sequence (arrows). (C) A magnetic resonance angiogram revealed severe stenosis of the right middle cerebral artery with reduced filling of the distal vessels (geen arrow). She was followed for 3 years and did not develop neurological symptoms

**Figure 5.**
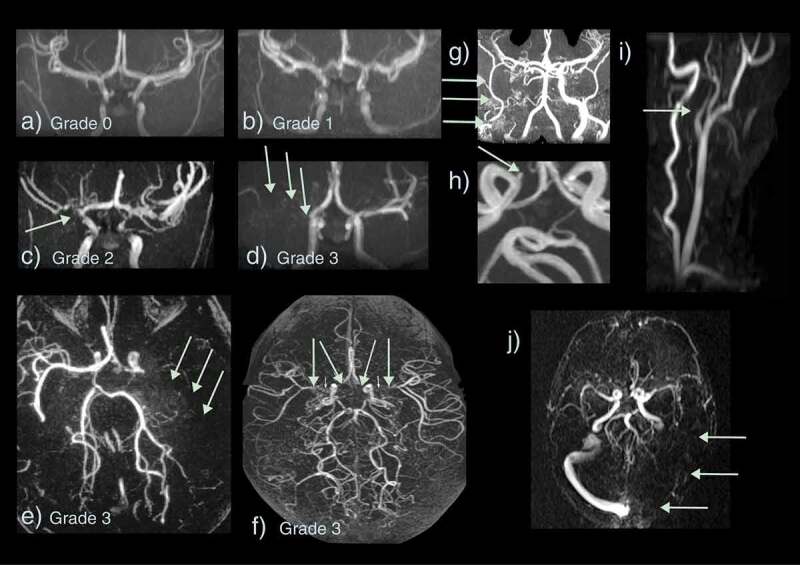
Vasculopathy on magnetic resonance angiography and venography (MRA/V) in sickle cell disease. Arteriopathy is graded as (a) 0 – none, (b) 1 – minor signal attenuation, (c) 2 – obvious signal attenuation but presence of distal flow, (d) 3 – signal loss with and without collaterals i.e. occlusion (e) Grade 3 – occluded left middle cerebral artery (MCA) (green arrows) with basal ganglia and posterior pial collaterals (f) Grade 3 – bilateral occluded terminal internal carotid arteries (ICA) and proximal MCAs with basal ganglia and pial collaterals visible (g) Grade 3 – occluded proximal right ICA with external ICA (green arrows) and skull base collaterals (h) Small aneurysm of the right cavernous ICA (green arrow) (i) Dissection of the origin of the left common carotid artery showing a typical string sign (green arrow) (j) Occluded left transverse and sigmoid sinus on MRV

For an acute ischemic focal neurological event, T2-fluid attenuated inversion recovery (T2-FLAIR) MRI is usually abnormal within a few hours, whereas diffusion-weighted images (DWI) can show ischemic regions within minutes, before irreversible infarction has occurred. Ischemic lesions appear hyperintense on raw DWI (Figure 2) and hypointense on quantitative maps representing the apparent diffusion coefficient (ADC; Figure 1), while T2-FLAIR changes may not be obvious for hours or days (Figures 1–4). Approximately three quarters of strokes occurring in people with SCD are ischemic, mostly in an arterial distribution (Figures 2–4) [1–4] associated with intra- and extracranial arteriopathy (Figure 5A-G,I), although venous thrombosis has been reported (Figure 5J) [1–4] and may be missed if venography is not performed in the acute phase. Without monthly blood transfusion, recurrence of ischemic stroke occurs in 67–90% of children with SCD [20]. The STOP studies demonstrated that Transcranial Doppler ultrasound (TCD) is a useful tool for screening and detection of patients at risk of stroke in the pediatric SCD population [1–4]. Since the STOP trial ended prematurely, due to the very large advantage in favor of blood transfusion for children with velocities >200 cm/s, screening and transfusion for at least 1 year have been recommended as standard care for pediatric SCD populations in the USA and the UK [1–4]. In children with abnormal TCD but normal MRA, i.e. no observable vasculopathy, non-inferiority of hydroxycarbamide was demonstrated in the TWiTCH trial, a primary stroke prevention trial for SCD children who had received at least 12 months of blood transfusion [21]. Recent epidemiological evidence suggests that there has been a parallel fall in the incidence of stroke in SCD and that screening and treatment strategy is cost-effective [22]. There are, however, well-described limitations, including the relatively low specificity of screening, the lack of research on the utility of screening in adult populations, the unclear evidence-base for management of children with both abnormal TCD and MRA, the burdensome nature of transfusion with complications, such as iron overload, and the continuing risk of progressive arteriopathy (Figures 2 and 5F,G) and stroke [23], as well as other neurological complications [1–4] (see below), in some patients.

### 2.3. Hemorrhagic stroke

**Figure 6.**
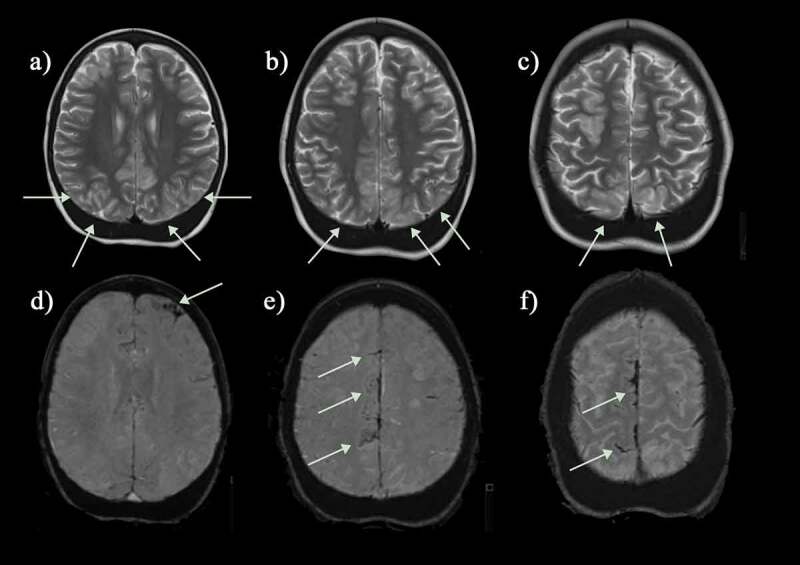
This teenager presented with sudden onset of blindness and seizures soon after acute chest syndrome. (a-c) There is occipital and frontal cortical and subcortical swelling on T2-weighted MRI, consistent with posterior reversible encephalopathy syndrome (green arrows) and (d-f) the susceptibility weighted imaging (SWI) revealed hemorrhage in the parafalcine and left frontal regions (green arrows). The magnetic resonance angiograms was normal. The patient returned to mainstream school but developed epilepsy

The remaining quarter of strokes in SCD are hemorrhagic (Figure 4, 6), although as they may cause sudden death, this is almost certainly an underestimate. Intraparenchymal, intraventricular, subarachnoid and occasionally subdural hemorrhages have all been described in patients with SCD [1–4] and may be detected using T1, T2, T2* (Figure 4) or susceptibility-weighted (Figure 6) MR sequences, as well as on CT. Cerebral hemorrhage in older patients is commonly related to aneurysm formation, detectable on MRA (Figure 5H) [25]. Aneurysms that rupture are typically located at the bifurcations of major vessels, particularly in the vertebrobasilar circulation. Intraparenchymal bleeding may be associated with large vessel vasculopathy, especially if moyamoya (Figure 2D) formation is present. Venous sinus thrombosis (Figure 5J) and reversible posterior leukoencephalopathy (Figure 6) may also be associated with hemorrhage. There are reports of epidural hematomata in the absence of significant head trauma in SCD [26,27], probably related to hypervascular areas of bone.

### 2.4. Other clinical presentations

Patients with SCD also have seizures or transient ischemic attacks (TIAs) with symptoms and signs resolving within 24 hours [1–4], although many of these individuals are found to have recent cerebral infarction or atrophy on qualitative MRI. In addition, acute coma, seizures and headache [28] are common in patients with SCD secondary to posterior reversible encephalopathy syndrome (PRES; Figure 6) [29,30], e.g., after acute chest syndrome (ACS), or in other settings where there is hypertension and/or immunosuppression [1–4]. There is a wide differential of alternative focal and generalized vascular and non-vascular pathologies, including intracranial hemorrhage, extensive infarction, central nervous system infections, and autoimmune encephalopathies, often distinguished using MRI techniques, with important management implications [1–4]. DWI may distinguish between infarction (Figure 2) and reversible phenomena, including edema, penumbral ischemia, PRES (Figure 6) [1–4] or small focal ischemic white matter lesions, termed acute silent cerebral ischemic events (ASCIE), e.g. after acute anemia with Parvovirus or splenic sequestration [31].

## 3. Qualitative imaging in steady-state settings

### 3.1. Covert or ‘silent’ cerebral infarction

**Figure 7.**
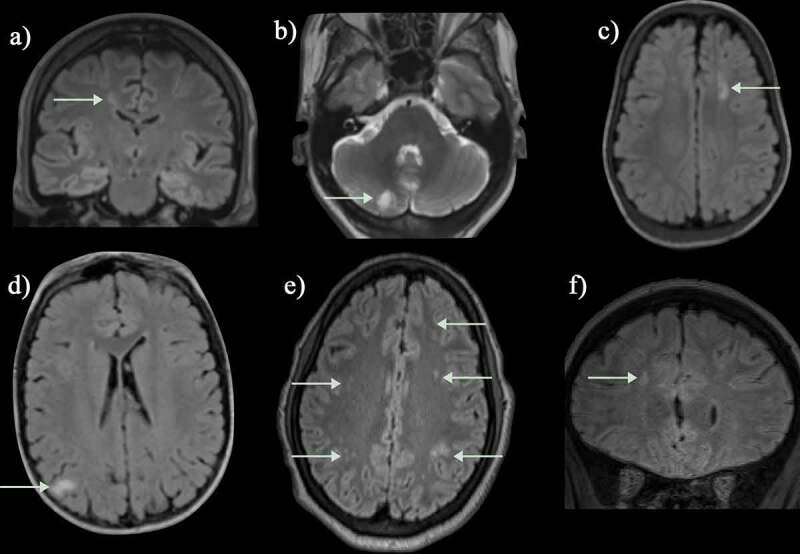
Silent infarcts in a group of asymptomatic children with sickle cell disease. (a) Coronal flair shows a watershed infarct in the right anterior cerebral artery/middle cerebral artery (ACA/MCA) territory (b) Axial T2-weighted image shows a mature cerebellar hemispheric infarct which could date back to birth (c) Axial FLAIR reveals a mature watershed infarct in the left ACA/MCA territory (d) A right parietal subcortical infarct on an axial FLAIR image. (e) Multiple scattered deep and subcortical white matter silent infarcts on axial FLAIR image. (f) Larger frontal deep white matter infarct of the right frontal lobe on coronal FLAIR image. The magnetic resonance angiograms were normal in all patients. FLAIR = fluid attenuated inversion recovery

**Figure 8.**
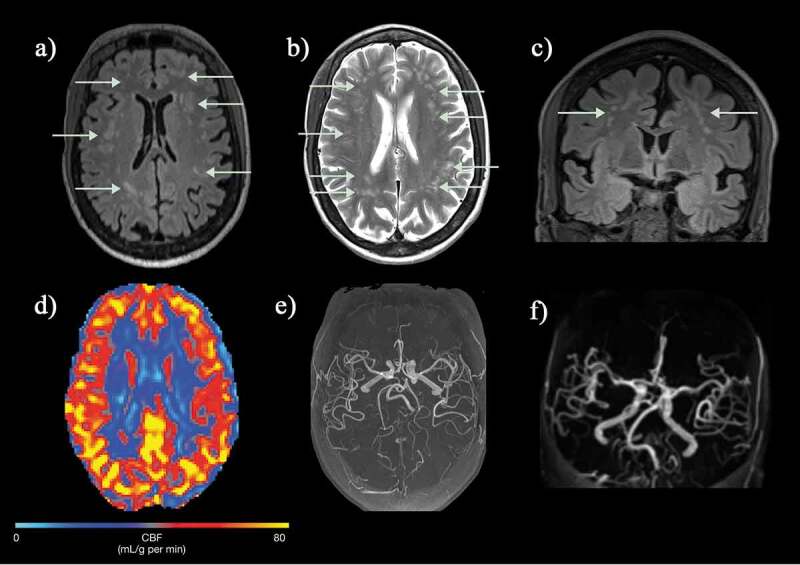
(a, b) Axial FLAIR and T2-weighted images and (c) coronal FLAIR images in a normally functioning 57-year-old female SCD patient with no neurological deficits who was scanned as part of a study and found to have widespread white matter lesions in the subcortical and deep white matter regions. (d) Cerebral blood flow map (CBF) map showed slightly reduced perfusion anteriorly and (e, f) intracranial MRA was entirely normal. FLAIR = fluid attenuated inversion recovery

As well as presenting with obvious acute neurological events, patients with homozygous SCA and sickle β-thalassemia, accumulate ‘silent’ cerebral infarction (SCI) from infancy through to adulthood (Figures 7 and 8) [32–34]. SCI is most commonly defined as a hyperintense region, visible in at least two planes on T2-FLAIR MRI, measuring at least 3 mm in children and 5 mm in adults [8], occurring in the absence of focal neurological symptoms. There is sometimes confusion regarding the terminology around SCI, with the radiological definition encompassing not only hyperintensities in white matter that are common in many populations including the healthy elderly (i.e. white matter hyperintensities) but also cortical hyperintensities that may occur unnoticed.

Many patients present with SCI without having had a clinically overt stroke, although it may be difficult to exclude subtle TIAs, headaches or seizures or other minor difficulties with motor function (‘soft neurological signs’) that may be missed without full formal neurological testing or assessment of medical history. There is a predilection for overt ischemic stroke and SCI in the borderzone areas of the brain demonstrated in several studies using T2-FLAIR MRI [35](Figures 7–8); the higher the field strength of the magnet and the older the patient, the greater the proportion of patients with SCI [36,37]. ASCIE may also be documented on DWI in steady-state settings [31] and appear to be four times more common than SCI, with only some later transitioning into SCI observable on T2-FLAIR [38]. The factors involved in determining this tipping-point remain unclear, but reductions in oxygen delivery, along with exhaustion of flow and oxygen reserves, have been proposed to play a role [16].

Cognitive difficulties are apparent across a number of domains. There is marked dysfunction in cognitive areas, such as executive functioning, processing speed, and attention, as well as full-scale intelligence quotient [4,39,40]. Although patients with SCD and clinical stroke or SCI appear to be more likely to have abnormal psychometric testing, deficits may occur also in patients with no observable pathology on qualitative MRI [39,40].

### 3.2. Vasculopathy

Anterior cerebral circulation vasculopathy is well recognized in patients with stroke in the context of SCD on digital subtraction contrast angiography [41], which shows the anatomy of the vessel wall. MRA may also visualize abnormal vessels [9–11,13,35,42–45], although this depends on disturbance to vessel flow or ‘turbulence.’ Data from non-sickle pediatric stroke suggest that large vessel disease is detected by MRA as well as digital subtraction angiography [46]. However, possible flow disturbances in SCD patients may exacerbate the technical challenges associated with the assessment of the severity of vascular disease using MRA. In a study of 21 children with SCD, 14 with previous stroke and 7 asymptomatic, the overall sensitivity of MRA in the posterior and anterior circulation was 50% and 81%, respectively. Sensitivity was higher (93%) for detecting occlusive lesions in the ICA and MCA than the ACA (54%), while specificity was 96%, 86%, and 100% for these vessels, respectively, [47]. Time-of-flight MRA may overestimate the degree and extent of stenosis in children when compared to cerebral angiography [48] due to intravoxel dephasing (turbulent flow) of the blood within normal vessels, particularly in SCD patients. Of note, intravoxel dephasing has been observed in patients with normal cerebral angiography who go on to develop large vessel vasculopathy, indicating that dephasing may represent high velocity or complex patterns of flow in the early stages of vascular disease [47]. There have, however, been no large prospective studies specifically examining whether intravoxel dephasing or ‘turbulent flow’ predict later development of large vessel vascular disease.

The prevalence of MRA abnormality (Figure 5) varies considerably; in addition to whether the population includes asymptomatic patients as well as those with stroke, this is in part related to the definition of abnormality [12]. Some studies have reported the degree of arterial stenosis, typically reporting >50% stenosis as abnormal [12]. Helton and her colleagues used an intracranial MRA vasculopathy grading scale in 150 scans, considering vessels as abnormal if there was stenosis/occlusion >5 mm across 20 segments in total, combined as grades 0–6 according to severity and number of abnormal vessels [10]. Although this allows understanding in the context of previous experience with digital subtraction angiography, unless surgery is contemplated, this invasive imaging technique is rarely used now for ischemic stroke, even in symptomatic patients with SCD, so there are few data for comparison. Alternatively, signal dropout or turbulence on MRA may be graded as none (grade 0), mild (grade 1), moderate (grade 2), or severe with or without collaterals (grade 3) (Figure 5) [11,13,24,45], or even as normal or abnormal [35]. The available data suggest that even mild abnormality is associated with tissue compromise [24,35]; this may be a useful research strategy given that studies comparing MRA with digital subtraction angiography would be unethical in asymptomatic patients.

Despite these concerns, MRA is used in protocols for screening asymptomatic patients as it is noninvasive. For MRA of the intracranial vessels, signal dropout in the distal internal carotid artery (ICA) and the proximal middle (MCA) and anterior cerebral arteries (ACA) is typical (Figure 5) [9–11,13,35,42–45]. Occlusion may be associated with the development of bypassing collateral vessels, known as moyamoya (Figure 2D), from the Japanese expression describing the angiogram appearing like a ‘puff of smoke’ [49]. In addition, it has recently become clear that patients with SCD may have extra-cranial vasculopathy, including dissection as well as narrowing or occlusion (Figure 5G,I), detectable on imaging of the neck [50,51], and that this is associated with SCI [51]. Tortuosity is relatively common (Figure 5I) [52] and ectasia of the basilar and intracranial circulations has also been documented and is associated with low hematocrit [53]. It is worth considering separately the vasculopathy literature in the context of clinical stroke, SCI, and screening for stroke risk in asymptomatic patients.

### 3.3. MRA-defined large vessel vasculopathy and ischemic stroke

The majority of SCD patients with stroke have a narrowing of the arteries of the Circle of Willis at the base of the brain [11,45], associated with cerebral infarction either in the middle cerebral artery territory or more characteristically in the superficial and deep borderzones between the anterior and middle cerebral artery territories. In the SWiTCH trial which randomized 161 children with SCD who had previously had a stroke to continue regular transfusion or to start hydroxyurea and phlebotomy, around a third had no stenosis on either side despite having previously had a stroke [10]. Children with low or uninterpretable TCD velocities had the worst stenosis and children who had stroke or transient ischemic attacks during the trial had substantial vessel stenosis and parenchymal injury; in one the vasculopathy had evolved from grade 0 to grade 5 in association with a stroke [10]. At exit, of 112 children, one had worse stenosis and one had a new SCI, both in the hydroxyurea and phlebotomy arm. Others have also found that in patients with SCD and stroke, MRA abnormalities progress even with chronic blood transfusion [23], particularly if there was more severe abnormality at baseline [54].

### 3.4. Screening asymptomatic patients

Transcranial Doppler is the mainstay of screening for stroke risk in children and there are data on MRA in those with abnormal velocities (>200 cm/sec). In the STOP study, MRA was undertaken at baseline in 100 patients with abnormal TCD, 47 in the transfusion arm and 53 in the standard care arm. MRA was normal in 75 patients, and demonstrated stenosis in 25 that was mild in 4 and severe in 21. In the standard care arm, 4 of 13 patients with abnormal MRA findings had strokes compared with 5 of 40 patients with normal MRA findings (p = 0.03) [55] Severe intracranial stenosis on MRA is more likely in children who have very high transcranial Doppler velocities, typically over 250 cm/second. Stenosis on MRA is also common in children with low TCD velocities and appears to predict stroke in this group. In view of these data suggesting that MRA abnormality is associated with stroke in children who are not transfused for high TCD velocities, the TWiTCH trial excluded those with MRA abnormality from switching to Hydroxyurea after a year of transfusion. However, progression of MRA abnormality in asymptomatic patients is much less common than in those who have had a stroke [54].

MRA can detect cerebrovascular disease in very young asymptomatic children [33]. In one study, MRA abnormalities were found in 3 out of 29 patients from 7 to 48 months of age [56].

As TCD is observer dependent and it is difficult to centralize training and over-reading, there is a case for the use of MRA as a research tool to understand the pathophysiology and the risk factors for initiation and progression of vasculopathy, and in randomized trials of management strategies to prevent progression [57]. For example, reticulocytosis is a common association with vasculopathy in SCD, probably in relation to chronic oxygen desaturation as well as hemolysis [11,13], and may not respond to regular blood transfusion [58]. In one study, vasculopathy was associated with mean prior values for hemoglobin, oxygen content, reticulocytes, and indirect bilirubin [13].

### 3.5. MRA-defined vasculopathy and silent cerebral infarction

Magnetic resonance angiography (MRA) abnormalities appear to be associated with SCI. In the Silent Infarct Transfusion Trial (SIT), MRI and MRA were available in 516 children; MRA was abnormal in 16% of children with SCI and 6% of children without [9]. SCI was nearly three times as common in those with vasculopathy in a study from Tanzania [13]. In a study visualizing the distribution of infarction using a heat map technique, large vessel vasculopathy in children was associated with cortical and widespread borderzone overt infarction, SCI, and atrophy [35]. Overt and silent infarcts were however also observed in the cerebral hemispheres without large vessel vasculopathy, implying that some may have a thromboembolic or chronic hypoxic-ischemic etiology [35]. Moreover, although when present, abnormal MRA appears to be associated with SCI, another study based on the SIT trial data shows that abnormal MRA is rare in children with SCI and normal TCD velocities (2%), further indicating that other factors are likely involved in the development of SCI [59].

### 3.6. MRA-defined vasculopathy and hemorrhagic stroke

Subarachnoid and intracerebral hemorrhage may occur, sometimes as a result of rupture of the fragile moyamoya vessels or of aneurysms usually located at the bifurcations of major vessels, most commonly in the posterior circulation. There are few data in patients with SCD, but MRA may detect aneurysms as incidental findings (Figure 5H), particularly at high field strengths [60] and may replace digital subtraction angiography for follow-up of treated aneurysms [61]. Other advanced MRI techniques may, however, be better for follow-up of untreated aneurysms [62,63]. Recent work has demonstrated that intracranial aneurysms are common in homozygous SCD, with a trend to being more common than the general population, particularly in women aged 30–39 years [25]. However, there appears to be no correlation between the occurrence of intracranial aneurysms and moyamoya syndrome [25].

### 3.7. Venous anatomy from magnetic resonance venography and susceptibility imaging

The anatomy of venous drainage appears to be different in people with SCD compared with controls, with reduced cortical venous conspicuity [64] and overall venular rarefaction and a different distribution (lower density of long and greater density of short venules) [65] on susceptibility-weighted imaging but greater dural venous sinus diameter [66]. There is no association with history of stroke [66] but the venular rarefaction may be associated with poor memory [65].

Additionally, there are several case reports of venous sinus thrombosis occurring in SCD patients, often in association with vaso-occlusive crisis, transfusion, or acute respiratory illness [67–69]. Venous sinus thrombosis (sinovenous thrombosis)(Figure 5J) may be associated with infarction or hemorrhage [70] and is probably underdiagnosed in SCD [69], although secondary edema may cause death. Anticoagulation is associated with reduced mortality and morbidity in the general adult population [70]. If neuroimaging is undertaken for acute neurological presentations, venography should be included, ideally MRV as the radiation dose for CT venography is high.

### 3.8. High-field scanning

The increased availability of high field strength MRI magnets (3 T, 7 T) over the more commonly used 1.5 T magnet, is likely to result in increased detection of brain abnormalities associated with SCD, including SCI, microhemorrhages, and perivascular spaces. Studies relying on lower field strength magnets (i.e. 1.5 T) with lower resolution sequences (i.e. 3–5 mm slice thicknesses) are by definition likely to miss some lesions meeting traditional SCI criteria, and there is evidence that lesion detectability increases with increasing magnet strength in SCD patients [36]. A substantial effect of high-field technology was demonstrated in a study of adult SCD patients scanned at 3 T using a 1 mm resolution sequence and 7 T using a 0.8 mm resolution sequence, where prevalence of SCI was found to increase from 50% at 3 T to 90% at 7 T [36]. Of note, recent high-field strength studies have suggested a relatively high prevalence of lesions also in healthy young adults [71,72], emphasizing the need for MRI studies to include control groups so that the clinical significance of any findings can be evaluated.

Improvements in MRA techniques at higher field strengths are also likely to result in improved evaluation of vessels and turbulent flow, but no comparative studies have been performed to date.

## 4. Quantitative imaging in steady-state settings

Advances in quantitative MRI are beginning to shed light on possible mechanisms of neurological and neurocognitive compromise in SCD patients with and without pathology detectable using qualitative MRI. There is accumulating evidence that overt stroke, SCI, and vasculopathy may represent only the ‘tip of the iceberg’ in terms of functionally significant brain abnormalities. Quantitative techniques offer several advantages over traditional qualitative approaches not only in terms of enabling measurement of parameters including brain volume, microstructural integrity, structural and functional connectivity, perfusion, and oxygenation but also in terms of minimizing issues related to subjectivity and statistical inference. Most quantitative techniques are however still in their infancy, with understanding of the specificity, validity, and reproducibility of quantitative imaging parameters still developing.

### 4.1. Morphometry

T1w MR images are optimized to primarily reflect differences in T1 relaxation between tissues. T1w imaging is widely used in conjunction with other methods for anatomical localization, as well as alone for morphometric analysis of parameters including gray and white matter density and volume, subcortical volumes, cortical thickness, and cortical curvature. Volumetric studies have consistently reported differences in brain volume in SCD patients. Pattern of change has varied between studies, which may be related to differences in precise processing pipelines and analysis techniques. Differences in the ages of the studied samples may also play a role. Studies in older children and adolescent SCD patients (>9 years) have consistently reported lower white-matter volume globally, and regionally in frontal, parietal, and temporal lobes, as well as in the corpus callosum, brainstem, and cerebellum [71,73,74]. In one study directly comparing radiologically normal children and adolescents with SCD and controls, patients showed bilateral decreases in white matter density across the corpus callosum and along the ventricles [73]. In a more recent study, white matter volume in SCD patients was 6.8% lower on the left and 8.1% lower on the right, with the areas affected including the corpus callosum, right brainstem, and right cerebellum, as well as the frontal, parietal and temporal lobes bilaterally, although the occipital region was relatively spared [71]. Other studies have also documented asymmetry with greater atrophy on the right side of the brain [75].

For gray matter volumes in late childhood and adolescence, findings are less consistent, with two studies finding few differences in gray matter volume globally or regionally [71,73], and one finding widespread differences in subcortical structures [75]. The latter study reported lower hippocampal, amygdala, thalamus, cerebellum, and basal ganglia volumes in SCD patients irrespective of SCI status, though the degree of volume reduction was greater in those with SCI [75]. Cortical morphometric findings in this age group have also been mixed, with two studies finding cortical thinning in regions with high metabolic activity in older children and adolescents with SCD [76,77], a third study finding no differences [71], and a fourth study finding increased posterior pericalcarine cortical thickness [78].

### 4.2. Predictors of brain volume

Older age and female sex were the strongest predictors of lower gray matter volume in one study [71]. However, for lower white matter volume, male sex, lower hemoglobin, and lower mean platelet volume were predictors, while the presence of SCI, hemoglobin S, fetal hemoglobin, and markers of hemolysis, such as cell-free hemoglobin and lactate dehydrogenase, were not [71]. A study of adolescents and young adults found that the majority of white matter structures in those with SCD and an anemic control group had reduced volumes compared with non-anemic controls after adjusting for age, sex and education level, suggesting an effect of anemia rather than sickle hemoglobin [79]. Another report from this group which included larger numbers of patients with SCD and other chronic anemias, such as β-thalassemia and spherocytosis, also emphasized the importance of anemia severity in predicting reduced white matter volume [80].

### 4.3. Developmental changes in brain volume

A pattern consistent with abnormal maturation was described in the only prospective longitudinal study of gray and white matter morphometry in young SCD children to date [81], which included patients and controls between the ages of 3 and 16 years. In this study, change in global gray matter in patients was best captured by a model of linear decrease with age, whereas change in gray matter in controls was best described by a quadratic model in which there was an initial increase in global volume, followed by a period of stabilization and slight decrease. Moreover, whilst change in global white matter in patients was best described by a model of linear increase, the rate of increase was approximately half of that observed in controls [81].

These results are consistent with a study tracking overall percent brain volume change over a three-year period in a subset of children with SCD enrolled in the SIT trial [82], which showed rates of decline beyond those observed in healthy populations, similar to those seen in elderly populations with leukoaraiosis. In the larger SIT trial dataset including 157 of the 196 participants [83], similar volume loss was demonstrated, with multiple regression analyses indicating that loss of volume becomes progressively worse with age. Regular blood transfusion for 3 years did not appear to arrest brain volume loss in either report [82,83].

While change in white matter volume over time has not been investigated in adults with SCD, linear decline in volume with age is consistent with the only morphometric study in adult sickle patients, where patients showed lower basal ganglia and thalamus volumes, and thinner frontal lobe cortex, compared to controls [84]. Murine studies have also found volumetric differences in gray and white matter volumes, including in subcortical structures [85,86]. Moreover, older SCD mice show neuropathologic changes in the hippocampus and cerebellum that are not seen in control mice, including shrunken neurons [86].

Taken together, these findings are indicative of significant abnormalities in brain size and growth that may begin early in development and persist into adulthood, irrespective of the presence of SCI. Discrepant findings between cross-sectional studies may be related to the inclusion of highly heterogenous samples in terms of age. Normal brain maturation is thought to involve synaptic formation, new cell generation, synaptic pruning, and myelination [87–89]. Disruption to any of these processes could delay the rate of maturation, and/or result in apparent or actual tissue loss. Of note, however, axons that have yet to be myelinated may have abnormal white matter signal on MRI, leading to volume averaging with gray matter. Due to this partial-volume effect, an apparent decrease in gray matter volume could in reality reflect an increase in myelination [88]. Even in longitudinal volumetric MRI studies, it can therefore be difficult to determine whether patterns of change reflect impaired growth (delay) or tissue loss (atrophy). Studies of normal brain maturation have also highlighted regional differences in patterns of change, with some longitudinal studies showing regions of progressive volumetric changes that continue into adulthood, without leveling off [90,91]. Modeling of change in overall gray and white matter volumes may therefore also mask regionally specific effects of SCD on volumetric development.

### 4.4. Brain volume and cognitive function

Voxel-based morphometry studies in children with SCD have found decreased gray matter volume in association with reduced IQ, and there is some evidence that cortical thinning predicts lower IQ [14]. In a recent study, lower volume at baseline in six gray matter structures (the left median cingulate gyrus, the right middle occipital gyrus, the left inferior occipital gyrus, the right fusiform gyrus, the right middle temporal gyrus, the right inferior temporal gyrus) predicted decline in scores on the Kaufman Brief Intelligence Test (K-BIT) assessed at yearly intervals for 2–4 years [92]. In adults, reduced white matter volume appears to predict lower full-scale IQ, mainly explained by difficulties in perceptual reasoning, in males but not females [80]. However, there is little evidence that the progressive decline in brain volume is associated with the progressive cognitive difficulties documented in SCD; for example, in the 32 SIT trial patients where the effect of change in brain volume on change in full scale IQ was examined, there was no evidence of a relationship [82]; the effect on cognition was not reported in the larger study [83]. It is unclear whether brain volumes change with treatment in SCD.

Overall, quantitative T1 imaging has mainly been used in observational cohort studies in SCD either in conjunction with other quantitative imaging techniques for anatomical localization purposes, or alone to investigate brain volumes and surface characteristics. While morphometry measures may provide useful endpoints in future clinical trials targeting anemia, their sensitivity to potential treatment effects remains unclear. Given the strong effects of age and development, such markers may hold most promise in trials in young homogeneous populations.

### 4.5. Microstructural integrity

Diffusion-weighted images (DWI) are optimized to detect the motion of water. Whilst water diffusion is isotropic in cerebral spinal fluid and gray matter, occurring more or less equally in all directions, diffusion in white matter is anisotropic, with more diffusion occurring parallel to neural tracts than in the perpendicular direction. In quantitative DWI, the degree and direction of anisotropy are modeled to make inferences about the integrity and architecture of the local tissue microstructure.

The average water molecular displacement is equivalent to the apparent diffusion coefficient (ADC) and is usually used in acute stroke imaging protocols. In diffusion tensor imaging, diffusion is modeled by a tensor, which can be visualized as an ellipsoid with three directions. From the tensor, several metrics can be derived: fractional anisotropy (FA), representing how far the tensor deviates from a sphere (i.e. the degree of anisotropy) and mean diffusivity (MD), axial diffusivity (AD), and radial diffusivity (RD), representing the average amount of diffusion across directions, in the principal direction, and in the perpendicular direction, respectively.

Whole-brain tract-based spatial statistics (TBSS) is a popular technique for examining DTI parameters at the core of major white matter tracts on a local voxel-wise basis. In SCD, TBSS studies have shown diffuse white matter damage in patients with SCA without SCI – see [14] for previous literature review. One UK study showed that FA was lower in the corticospinal tract and cerebellum in children with SCA compared to controls, while in widespread areas, MD and RD were higher [93]. There was a significant relationship between lower daytime oxygen saturation and higher RD in the anterior corpus callosum, with a trend level relationship in the same direction for hemoglobin [93]. A study of 83 UK children and young adults with SCA, 37 with SCI, focussed on the relationship between differences in DTI parameters and processing speed, as full-scale IQ was not lower in patients with SCA once processing speed was accounted for. The authors found that reduction in processing speed was associated with reduced FA across the internal capsule and corpus callosum and increased MD and RD across widespread regions, particularly posteriorly in the posterior corona radiata and the splenium of the corpus callosum [94]. A recent U.S. study examining specific white matter tracts similarly observed reduced integrity in SCD patients, with decreases in FA in the corpus callosum significantly associated with measures of processing speed, working memory, and executive function [95]. A fourth study in Tanzanian children with SCD found that, compared with those without SCI, those with SCI exhibited increased RD in multiple regions [24]. MD and AD were higher when hemoglobin was lower. Compared with SCA patients without vasculopathy, patients with vasculopathy exhibited reduced FA in widespread regions, including the anterior longitudinal fasciculi, corpus callosum, internal capsule, corona radiata, and corticospinal tracts [24]. Interestingly, the posterior cerebral arterial territory had a higher mean MD and mean RD than the anterior and middle cerebral arterial territories, although no patient had vasculopathy in vessels supplying this area [24].

To date, no studies have investigated changes in DTI parameters over the course of development in SCD patients, though they are known to change with age in healthy populations. There are also few data on the effect of treatment, although data from other clinical populations indicate that DTI parameters might serve as useful endpoints in randomized controlled trials [96]. A study looking at skew and kurtosis of global white matter MD histograms in adolescents with SCA and controls found that untreated patients with SCA had the highest skew and kurtosis, while those with SCA who were on hydroxyurea had intermediate values closer to the controls [97].

Whilst DTI is the most widely used technique for assessing tissue microstructure, it suffers from several limitations, including parameter confounding by crossing fibers and fiber orientation. DTI parameters are also not specific to particular microstructural elements of white matter, with the changes observed in SCD patients potentially reflecting demyelination or a failure to myelinate, reduced axon density, reduced axon coherence, and/or changes in tract volume, iron content, and/or water content. As for volumetric parameters, modeling of change in diffusion parameters across white-matter globally may mask regionally specific effects. Other advanced techniques for DWI data, including automated tract segmentation techniques [98], fixel-based analysis [99], and diffusion kurtosis imaging [100] have yet to be applied in this population. In summary, as for quantitative T1 approaches, diffusion parameters have primarily been used in observational studies to date. While they might serve as functionally significant endpoints in future clinical trials targeting anemic or hypoxic exposure, their sensitivity to treatment effects in SCD patients remains unclear. Again, given documented age-effects in other populations, DTI parameters may hold most promise as endpoints in trials with young, homogenous samples.

### 4.6. Quantitative susceptibility mapping (QSM)

Quantitative susceptibility mapping (QSM) techniques are optimized to detect and quantify tissue magnetic susceptibility, which is strongly correlated with iron content. Whilst brain iron accumulation may be of particular concern among chronically transfused SCD patients, increases in brain iron have been described in other conditions with ischemia-reperfusion injury, hypoxia, and vascular damage, all of which may be relevant in non-transfused SCD patients. There have however been relatively few QSM iron studies in this group.

In a study in 26 asymptomatic SCD adolescents and adults (5 receiving chronic transfusion, 2 with a history of chronic transfusion), iron content appeared to be greater in the putamen, substantia nigra, and red nucleus in the SCD group compared with healthy age and race-matched controls [101]. Although the sample was mixed with a minority receiving transfusion, brain iron levels were found to be independent of somatic iron burden, indicating that other factors may be at play.

Combining patients with SCD and controls, the authors described an increase in susceptibility with age in the substantia nigra, red nucleus, and dentate nucleus, as originally documented pathologically [101,102]. Whether iron increase plays a role in aggravating white-matter microstructural damage and cognitive impairment in SCD, and/or in accelerating neurodegeneration, are open questions. Overall, whilst the available evidence on QSM-based iron deposition suggests some promise as a potential biomarker of cerebral injury in SCD, much more work is required to assess associations with risk factors, other markers of tissue injury, and cognitive outcome.

In recent years, interest has also grown in the potential for QSM and other quantitative susceptibility techniques to provide estimates of oxygen metabolism, as described in the section on cerebral oxygenation below.

### 4.7. Structural and functional connectivity

Cognitive functions are thought to be sub-served by distributed parallel neural networks, including the somatosensory network and the default mode network (DMN); for the latter, connectivity is maximum at rest and is suspended during tasks. These can be modeled using quantitative MRI by reconstructing macroscopic structural and functional connectomes, based on DTI and the Blood Oxygenation Level-Dependent (BOLD) signal, respectively. Observational studies, to date, have mainly looked at differences in functional connectivity from resting-state functional MRI data (rsfMRI), e.g. amplitude of low-frequency fluctuations (ALFF) [79] or independent component analysis [103] between patients with SCD and controls and associations with hemoglobin, hemoglobin F, oxygen saturation, cognition and pain [79,103–111].

In 40 children with SCD aged 4.6 to 15 years, and 16 controls aged 5–15 years, Colombatti found increases in functional connectivity in the default mode network in those with SCD, particularly in those with verbal IQ <70 [104]. Connectivity was also higher in those with oxygen saturations of less than 97%, but there was no relationship with hemoglobin [104]. They suggested that the increase might be a compensatory mechanism but did not include measures of cerebral blood flow or metabolism. Another rsfMRI and EEG study observed decreased activity in the DMN and executive control network at rest in 15 SCD patients aged 16–38 years compared with 15 young adult controls [106]. However, in a study of adolescents and young adults with and without SCD, functional connectivity was only abnormal in 4 of 27 of those with SCD, in association with anemia and white matter hyperintensities [107]. The same group compared 20 youth with SCD (12 chronically transfused, 7 on hydroxyurea; none with MRA abnormality), 12 with other anemias (11 transfused), and 19 healthy controls (AA, AS), and found differences in connectivity (ALFF) in various brain regions, including the anterior cingulate cortex, insula, precuneus and medial superior frontal gyrus [79]. SCD patients had increased connectivity compared with other anemias in the medial orbitofrontal and anterior and posterior cingulate cortex but decreased connectivity in the frontal pole (associated with white matter hyperintensities and with reduced verbal fluency and cognitive flexibility from the Delis–Kaplan Executive function system), medial superior frontal gyrus (associated with white matter hyperintensities) and cerebellum. They did not include measures of pain but suggested that abnormal activity in these areas could be associated with the dysautonomia (decreased parasympathetic and increased sympathetic activity) as an alternative to reflecting previous exposure to pain, since there was no difference in those transfused, in whom pain was minimal, and the untransfused.

Acute pain secondary to vaso-occlusion is the cardinal symptom of SCD, but daily questionnaire data suggest that chronic neuropathic pain is increasingly important from childhood onwards [111–113]. This may be related to central sensitizing mechanisms which in turn may lead to alteration in the connectivity of networks measurable using fMRI. In a study of 25 young people with SCD aged 12–25 years stratified by pain burden, Darbari and colleagues found increased functional connectivity between cingulate cortex and DMN structures, considered a pronociceptive (pro-pain) network, in the high pain group (n = 8 patients with >3 hospitalizations for pain in the previous year) whereas connectivity was greater in antinociceptive structures in 14 with less pain [105]. The importance of posterior fossa structures, such as the cerebellum, the peri-aqueductal gray matter, and the locus coeruleus in the midbrain in pain processing was originally suggested in resting-state EEG data, where functional connectivity was stronger between the cerebellum and the periaqueductal gray matter, which is involved in inhibition of pain, and connections to pain processing areas were less [106], although pain was not assessed. Bhatt et al. showed that SCD patients had increased connectivity between the left locus coeruleus and the left dorsolateral prefrontal cortex compared with anemic controls, after accounting for the use of chronic transfusion therapy [108]. There was a trend for the three with SCD diagnosed with chronic pain on clinical grounds, e.g. use of words used to describe neuropathic pain, non-response to opioids, to have greater connectivity between the left locus coeruleus and the left dorsolateral prefrontal cortex compared with the 15 without chronic pain [108]. In 32 adults with SCD compared with 10 controls, Karafin et al. showed reduced connectivity for the peri-aqueductual gray matter with the anterior cingulate cortex but increased connectivity with the occipital gyrus and the parietal lobe [110]. Comparing the 7 with chronic pain, defined as disease-related pain on ≥3 days a week for 6 months, with the other 25 SCD patients, there were differences in connectivity between the peri-aqueductal gray matter and various brain regions, some part of the DMN: 13 sensory processing areas, 5 motor processing areas, 7 areas involved with processing emotion, and 5 areas involved in memory [110]. Compared with those without chronic pain, those with chronic pain had reduced connectivity between the peri-aqueductal gray matter and nine brain regions, including components of the DMN, suggesting compromise of the network involved in introspection when the brain is not actively involved in a task. Data from all these studies require extension across age groups with much larger numbers and better diagnosis of chronic pain, autonomic function, and cognitive compromise.

There are a few studies of task-based fMRI alongside resting state data, in part because the assumptions on which the calculation of the BOLD effect is based may not be valid in anemia, although differences between patients might still be clinically important. Using a black and white checkerboard with color reversal for 2 or 16 seconds as the visual stimulus, Zou compared primary visual cortex activation in 23 children with SCD aged 5 to 18 years and 21 controls with medulloblastoma [114]. BOLD activation occurred in all the controls but was not seen in 6 patients with SCD, including the two who were not on hydroxyurea; these patients had lower hemoglobin and HbF and higher absolute reticulocyte counts than the other 17 SCD patients [114]. The amplitude of the BOLD signal was lower in the SCD patients than in the controls for both durations and was positively correlated with hemoglobin and basilar artery diameter, an index of cerebral blood volume, as well as cognitive performance on the Wechsler Abbreviated Scale of Intelligence (WASI), but was not related to baseline cerebral blood flow [114,115]. The default mode network activated during rest in 24% of controls with medulloblastoma, but none of the SCD patients [114]. Sun et al. presented a word stem paradigm visually to 13 controls and 13 children with SCD, 3 of whom showed no activation while the other 10 had activation that was quantitively indistinguishable from 10 controls, although qualitatively it was more extensive in the SCD patients [109]. However, for deactivation to default mode, there were weaker and less extensive responses in the medial parietal cortex and the right angular gyrus and there was no response in the left auditory cortex in controls [109]. Another study of SCD patients aged 15–30 years compared with controls at rest and in response to an acute painful stimulus found hypo- and hyperconnectivity between various structures in the somatosensory, salience and default mode networks, which were more different in the resting state, consistent with an effect of chronic sensitization to pain [103].

To date, there have been no structural connectome studies in SCD to consider alongside the seemingly discrepant functional connectome findings. Given that rsfMRI and task-based fMRI are based on BOLD, which derives contrast from the differential properties of deoxygenated and oxygenated hemoglobin, anemia and hemodynamic stress may confound the signal in SCD patients. In other words, it is possible that reported differences in BOLD responses are a reflection of differences in blood rheology, oxygenation, and cerebral hemodynamics rather than neuronal responses. Although there are reports indicating minimal differences in BOLD responses between SCD patients and controls [106], further validation work is required with larger and more heterogenous samples. In summary, rsfMRI and task-based MRI measures have, to date, been used in observational cohort studies, with mixed results. However, with further validation and understanding of neurovascular coupling in SCD patients, measures of functional connectivity may eventually hold promise as endpoints in trials assessing treatments for chronic pain, and potentially cognitive outcome.

### 4.8. Perfusion

Perfusion MRI has the longest history of quantitative MRI in SCD. The overwhelming majority of studies have shown elevated global cerebral blood flow in both children and adults (CBF) [14], inversely related to hematocrit and arterial oxygen content. Elevated global CBF may be both a response to and a risk factor for cerebral hypoxia [116]. Studies have shown a reduction of global CBF following treatment with hydroxyurea [116], transfusion – more in children than adults [117], and bone marrow transplantation [118,119] Strouse *et al*. [120] found a strong inverse correlation between global CBF and both full-scale IQ and performance IQ.

Perfusion MRI techniques, either traditionally after injection of a paramagnetic contrast agent (*e.g*. gadolinium) or noninvasive magnetic ‘tagging’ of water molecules in arterial blood, have found abnormalities both globally and regionally, with findings indicative of a ‘perfusion paradox’ in children as well as adults [16]. In patients with chronic cerebrovascular pathology and stroke, dynamic susceptibility contrast MRI (DSC-MRI) has shown focal areas of reduced CBF and prolonged mean transit time in the affected regions corresponding to stroke-like lesions [116,121–123], as well as hemispheric asymmetry of signal [124]. Recent studies using arterial spin labeling (ASL) have had mixed results; some studies confirm elevated global CBF [125–127], while others find no differences between patients and controls [128]. Although several studies suggest that global white matter CBF is elevated in SCD patients [116,129,130], the elevation appears to be lower than that observed for gray matter, and may be insufficient to maintain oxygen delivery regionally [131]. There is evidence that CBF in the borderzone regions is disproportionately reduced in SCD patients, going beyond the watershed effect alone [131].

The use of ASL for CBF quantification is preferable to DSC-MRI as there is no need for injection of a contrast agent or ionizing radiation. While reference ranges for ASL-based CBF have been reported in the SCA population [132,133], studies have widely differed in acquisition and processing. CBF quantification depends on the T1 value of blood [134], which is assumed in some studies but might be more accurate if corrected for the individual’s hematocrit [133], or if measured directly using T1 MRI [134]. ASL-based estimations of CBF are also heavily contaminated by noise and partial volume effects.

There are further technical challenges associated with the application of ASL in hyperemic subjects, including labeling efficiency [135], which may be reduced, and venous outflow [136]. To combat these challenges, some authors have advocated for correction for labeling efficiency, along with the employment of two-compartment models, but further work on the validity of ASL kinetic models in SCD patients is required. Acquisitions with multiple inflow times [137,138] do not require prior assumptions about the necessary delay for the fully labeled bolus of blood to arrive and allow for further characterization of hemodynamic behavior within a voxel, though there have been relatively few studies in this population [137]. Overall, whilst further validation and optimization work would be beneficial, ASL-based estimation of CBF is widely used as a biomarker of hemodynamic stress in both observational studies and treatment trials in SCD patients, with several studies demonstrating sensitivity to change.

### 4.9. Combined diffusion and perfusion studies

Using gadolinium as a tracer, one study found perfusion/diffusion mismatch in areas seen as normal in T2-weighted images [121], suggesting CBF was reduced but not enough for cytotoxic edema and tissue death. Similarly, a combined ASL perfusion/diffusion study [116] found abnormal appearing white matter, described as leukoencephalopathy as well as SCI, had decreased CBF and also decreased FA.

### 4.10. Cerebrovascular reserve and autoregulation

When combined with a vasodilatory stimulus, quantitative MRI techniques including BOLD and ASL may be used to measure cerebrovascular reserve (CVR) capacity. Whereas the normal response is to increase CBF in response to a vasodilatory stimulus, such as hypercapnia, studies based on these techniques in SCD children and adults have demonstrated a reduced or even negative response [77,138], related to the anemia [72,138] and the resultant adaptive vasodilatation. Reduced CVR, alone or in combination with steal, may render the watershed regions particularly vulnerable to hypoperfusion in settings where there is increased metabolic demand (e.g. infection, pyrexia, seizures) or an acute drop in CaO_2_ (e.g. chest crisis with acute anemia and hypoxia) [139] which are not uncommon in SCD. There is evidence for an association between reduced CVR and cortical thinning in patients with SCD, particularly in regions with a high metabolic rate [77]. CVR capacity is an established biomarker of overt stroke risk in other populations with hemodynamic stress, including those with carotid artery stenosis [140]. Longitudinal studies are required to determine whether CVR capacity is similarly predictive of risk of stroke, SCI, or microstructural integrity in SCD populations.

### 4.11. Cerebral Oxygenation

Several quantitative MRI methods have emerged for assessing cerebral oxygenation noninvasively. In SCD populations, interest has grown in the potential for MRI estimates of oxygen delivery (CBF * arterial oxygen content) and oxygen extraction (arterial oxygen content – venous oxygen content/arterial oxygen content) to improve neurological risk prediction, particularly for stroke [16]. However, most current oxygen-sensitive MRI-techniques rely on calibration models, which may be invalid in conditions such as SCD where alterations in blood rheology and hemodynamics may challenge assumptions.

T2-oximetry methods, including T2-relaxation-under-spin-tagging (TRUST), are widely used for estimating venous oxygen saturation [141], and are based on the principle that the transverse relaxation (T2) of blood is dependent on its oxygenation saturation. T2-oximetry methods have revealed changes in venous saturation in SCD patients, but yield diametrically opposing results depending on whether the calibration model is based on bovine-hemoglobin [142], hemoglobin-A [143], or hemoglobin-S blood, with no consensus on model validity. Whilst prior studies in adult SCD patients using models based on bovine-hemoglobin and hemoglobin-A calibrations [142,144,145] indicate decreased global venous saturation (consistent with higher OEF), studies using models based on hemoglobin-S [127,145], indicate increased global venous saturation (consistent with lower OEF). Using individual T2 calibrations, a recent study found no difference between global venous saturation in adult SCD patients and healthy controls [146].

Venous saturation can also be estimated using susceptibility-based techniques, which take advantage of the susceptibility difference between deoxygenated and oxygenated hemoglobin to measure blood oxygenation. Therefore, the obtained voxel-based mean susceptibility also includes the contribution from the deoxygenated hemoglobin in capillary blood. Moreover, whereas T2-oximetry methods only provide an estimate of global venous saturation from the T2 relaxation within a few voxels, susceptibility-based techniques can provide estimates throughout the venous vasculature. In children with SCD, susceptibility-based studies have indicated higher oxygen extraction, particularly in watershed regions of the deep white-matter where CBF is lower and the density of SCI is higher, potentially indicative of a compensatory mechanism [130]. Studies from the same group have also demonstrated reductions in OEF following transfusion and hydroxyurea therapy, with watershed regions continuing to exhibit ‘at-risk’ regions of elevated OEF [147], which appear to be relatively larger in patients treated with hydroxyurea compared to those on transfusion regimens [148]. However, also for susceptibility-based techniques, findings in SCD patients have been mixed, with a recent study indicating elevated venous saturation in children with SCD, consistent with reduced oxygen extraction [149]. While interesting, the treatment findings do not speak to the direction of the OEF findings, as it is possible that diametrically opposing results would be yielded with a different technique and/or model.

Differences in estimates of venous saturation have led to similar differences in estimates of global cerebral oxygen metabolism. For all current oxygen-sensitive MRI techniques, questions remain around the validity of the underlying assumptions, underscoring the need for further work comparing noninvasive MRI methods with each other, and with positron emission tomography techniques and/or gold-standard jugular vein catheterization. Other important questions for future research include the extent to which OEF changes are spatially consistent or heterogenous, and whether there are age-related changes in the direction of effects. As has been observed in populations with carotid artery occlusion [150], with further validation, OEF may hold promise as a future biomarker of stroke risk in SCD populations.

## 5. Expert opinion

In summary, MRI has a long history in SCD and continues to be a useful tool in screening, management, and research. In clinical settings, qualitative MRI plays a vital role in the detection and diagnosis of acute neurological complications including ischemic and hemorrhagic stroke, as well as chronic vasculopathy. As clinical sequences become faster and higher resolution, the presence of abnormalities may appear to increase. In order to better understand the clinical significance of more subtle radiological abnormalities detected using higher resolution MRI, it is important that future studies include healthy control samples well matched for age and other demographic characteristics (i.e. ethnicity, socio-economic status).

In research settings, structural quantitative MRI studies have revealed that the total extent of brain injury may go beyond stroke, SCI, and vasculopathy in SCD, with progressive volumetric and microstructural white-matter abnormalities that may be more prevalent, widespread, and potentially also more functionally significant. Hemodynamic quantitative MRI studies are beginning to shed light on potential underlying mechanisms, including hemodynamic stress, and an exhaustion of vascular flow and oxygen reserves. Quantitative MRI findings have however been mixed, identified structural changes are nonspecific, and further studies on the association between qualitative radiological findings, and quantitative structural and hemodynamic findings, are required.

A major advantage of quantitative multi-modal MRI approaches is that they provide continuous measures that are more normally distributed, and more powerful to work with statistically. Longitudinal quantitative multi-modal techniques may also help in overcoming another major challenge around the use of qualitative radiological techniques, which is that insults (e.g. silent cerebral infarction) are rarely detected at the time they occur. Such studies may, for example, help identify factors involved in determining transition from reversible ASCIE observable on DWI to chronic silent cerebral infarction observable on FLAIR. They may also shed light on important questions around whether acute lesions are reversible, or whether they produce permanent damage that is below the resolution of qualitative MRI. Finally, such studies may help in improving understanding of any potential age-dependent and/or developmental effects.

Along with larger and more comprehensive longitudinal studies and randomized controlled trials with structural and cognitive endpoints, one of the most pressing needs for patients with SCD and the clinicians tasked with treating them, is better methods for predicting individual risk of neurological morbidity. This would enable ongoing monitoring of risk so that treatment is not necessarily lifelong, as well as selection of patients who are sufficiently high-risk for burdensome and costly treatment. At present, risk-benefit analyses are subject to substantial uncertainty in SCD. Risk stratification of patients unlikely to need prevention for primary stroke, or likely to fail secondary prevention, from those likely to benefit from primary or secondary stroke prevention, are pivotal next steps that would increase the benefit-to-burden ratio. In future, early identification of the risk of structural abnormalities and associated cognitive impairment may enable implementation of preventative strategies before delay/decline occurs. In addition, with the adaptations to chronic anemia in SCD, which may increase the risk of some complications while reducing others, as promising treatments are developed, screening with MRI techniques could rapidly ascertain whether prevention of progressive brain damage is possible without causing it. To this end, novel quantitative hemodynamic and oxygen-sensitive MRI techniques hold promise, though as with all of these novel techniques, significant validation work is required.

## Article highlights

- Neurological pathologies detectable on radiological MRI, including vasculopathy and overt and silent stroke, are among the most common, but poorly understood, manifestations of sickle cell disease (SCD).
- Whilst there are radiological evidence-based strategies for stroke prevention in children with SCD, the specificity of screening is poor, treatment is often burdensome, and many patients continue to suffer progressive vasculopathy and/or recurrent insults.
- In recent years, evidence has emerged indicating that pathologies observable on radiological MRI may represent only the tip of the iceberg in terms of the causes of neurological morbidity in SCD.
- Quantitative MRI studies have identified cerebral hemodynamic, metabolic, volumetric and microstructural abnormalities that may be more prevalent, widespread, and potentially also more functionally significant, but no risk-stratification or treatment approaches exist for these pathologies.
- With further validation, quantitative MRI techniques may hold promise not only in providing clinically significant endpoints for trials of novel treatment approaches but also in improving risk stratification and individualized treatment options for precision medicine.
